# Delayed Presentation of a Giant Ascending Aortic Aneurysm following Aortic Valve Replacement

**DOI:** 10.1155/2009/740247

**Published:** 2010-02-09

**Authors:** Tugrul Göncü, Mustafa Sezen, Hasan Ari, Osman Tiryakioglu, Gündüz Yumun, Senol Yavuz

**Affiliations:** ^1^Department of Cardiovascular Surgery, Bursa Yüksek Ihtisas Education and Research Hospital, Bursa, Turkey; ^2^Department of Cardiology, Bursa Yüksek Ihtisas Education and Research Hospital, Bursa, Turkey

## Abstract

Giant ascending aortic aneurysm formation following aortic valve replacement is rare. A 28-year-old man who underwent aortic valve replacement with a prosthetic valve for aortic regurgitation secondary to congenital bicuspid aortic valve about 10 years ago was diagnosed with a giant ascending aortic aneurysm about 16 cm in diameter in follow-up. The aneurysm was resected leaving the functional old mechanical prosthesis in place and implanted a 34-mm Hemashield woven graft, associated with the left and right coronary artery button implantation. Histological findings of the aortic aneurysm wall showed cystic medial necrosis. The postoperative course was uneventful and postoperative examination demonstrated good surgical results.

## 1. Introduction

Patients with bicuspid aortic valve are at increased risk for aortic complications and that aortic valve replacement does not prevent progressive aortic dilatation [1, 2]. In these patients, large ascending aortic aneurysm formation following aortic valve replacement is very rare but serious complications with the possibility of rupture or dissection warrant surgical intervention. Giant aneurysm is defined as an aneurysm more than 10 cm in diameter [3]. We present a giant ascending aortic aneurysm about 16 cm in diameter which developed after AVR due to bicuspid aortic valve. The treatment of these aneurysms is a technical challenge and carries a high morbidity and mortality [3].

## 2. Case

**Figure 1:**
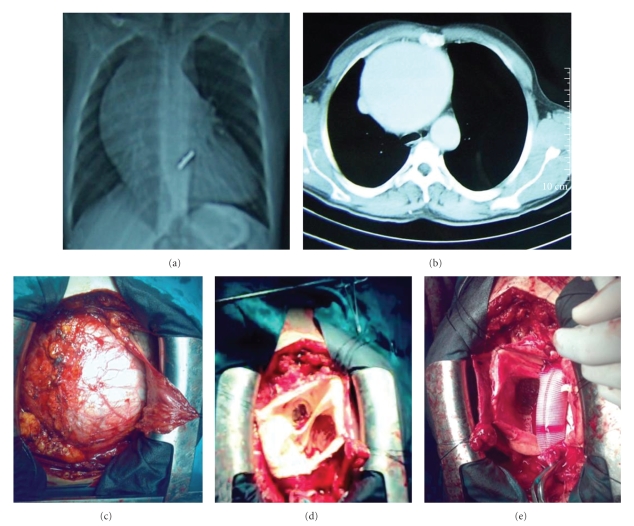
(a) Chest X-ray imaging showing as an aortic enlargement. (b) Spiral thoracic computed tomography imaging showing as a 16-cm aneurysm of ascending aorta. (c) The aortic aneurysm was occupying most of the space in the pericardial cavity. (d) and (e) The aneurysm of the ascending aorta was resected and implanted with a woven graft associated with the left and right coronary artery button implantation.

**Figure 2:**
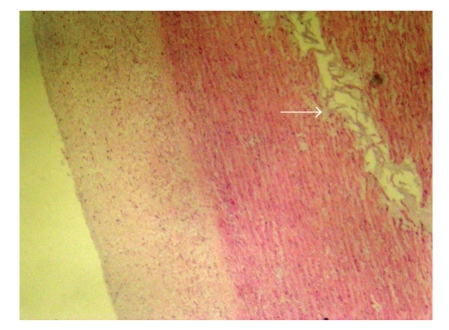
Pathological findings are demonstrating typical cystic medial degenerative changes. Histologically, it had the appearance in the media of “cystic spaces” filled with mucoid materials (arrow). (Hematoxylin & Eosin, original magnification × 100).

A 28-year-old man with severe aortic regurgitation underwent aortic valve replacement with a mechanical prosthetic valve about 10 years ago. At the time of this operation the ascending aorta was slightly dilated and measured as 3.4 cm. An ascending aortic aneurysm was suspected with chest X-ray in routine follow-up of the patient who had not been controlled until that time (Figure 1(a)). Echocardiography and chest-enhanced computed tomography revealed a giant ascending aortic aneurysm about 16 cm in diameter with intact aortic arch (Figure 1(b)). Prosthetic valve function and other cardiac structures were assessed as normal with two-dimensional and color Doppler examination. An elective operation was planned for the aortic aneurysm. The operation was performed under cardiopulmonary bypass, established by cannulation of the right femoral artery and right atrium via the right femoral vein. Cardiopulmonary bypass was started before sternotomy to decompress the aneurysm. Chest was opened with a median resternotomy. A giant ascending aortic aneurysm was occupying most of the space in the pericardial cavity, with the heart lying posteriorly (Figure 1(c)). The aortic arch was not found to be involved. After careful dissection of the aneurysm we were able to cross clamp the aorta proximally to the brachiocephalic trunk. After cross-clamping, the aorta was opened and cardioplegic solution was infused into each coronary artery. The previously implanted valve prosthesis was intact and assessment of valve functions was normal. Aneurysm of the ascending aorta was resected leaving the functional old mechanical prosthesis in place and we implanted a 34-mm Hemashield woven graft (Meadox Medicals Inc, Oakland, NJ, USA), associated with the left and right coronary artery button implantation. Distal anastomosis of the aortic graft was performed under aortic cross clamp (Figures 1(d) and 1(e)). Aortic clamping time was 117 minutes. Weaning from cardiopulmonary bypass and the postoperative course was uneventful. The patient was discharged without complication 10 days after surgery. Marfan syndrome was clinically excluded. Histological findings of the aortic aneurysm wall showed cystic medial necrosis (Figure 2).

## 3. Discussion

Aortic complications occurring after aortic valve replacement (AVR) include aortic dissection, ascending aortic aneurysm, aortic root aneurysm, and pseudoaneurysm [1–4]. To our knowledge, current case is one of the largest true ascending aortic aneurysm published in the literature which developed after AVR. 

The presented case had been operated before for severe aortic regurgitation secondary to congenital bicuspid aortic valve. Bicuspid aortic valve (BAV) is one of the most common congenital disorders involving the heart valves, with a prevalence ranging from 1% to 2% of the entire population [1]. Data from several groups [2, 4, 5] demonstrated that patients with BAV are at increased risk for aortic complications, even late after aortic valve surgery, because of histopathological changes in the ascending aorta, predisposing one to aneurysm development and dissection. There is evidence to suggest an intrinsic smooth muscle abnormality which leads to a higher rate of cystic medial degeneration in those patients with congenital bicuspid valve disease [4–6]. In the present case, the histopathological findings of aneurysmal tissue were consistent with medial cystic degeneration. Association between BAV and cystic median necrosis of the aorta relates to a common embryologic origin of the aortic valve and of the aorta itself [1, 5]. The aorta of a patient with a BAV contains less elastic tissue as compared to a normal (tricuspid) aortic valve [2]. Specifically, patients with a BAV have thinner elastic lamellae of the aortic media and greater distances between the elastic lamellae than in patients with a normal aortic valve [1, 2]. Patients with BAV are at increased risk for aortic complications and that aortic valve replacement does not prevent progressive aortic dilatation [2, 6]. In a study of 35 patients (20 patients with BAV) undergoing aortic valve replacement with aortic diameters greater than 40 mm the authors noted that 5 patients had a subsequent aortic event. The recommendation arising from this paper is for all patients undergoing AVR with an ascending aorta greater than 40 mm should have their ascending aorta replaced at the same time [6]. Borger et al. [7] suggested that patients undergoing operations for BAV disease should be considered for concomitant replacement of the ascending aorta if the diameter is 4.5 cm or greater. Recent guidelines from the American College of Cardiology/American Heart Association confirm such a strategy [8]. However, not all patients with BAV will develop aortic dilatation over time. Nistri et al. [9] observed a 52% prevalence of aortic dilatation in a series of young patients with normally functioning BAV.

Based on the law of Laplace, wall tension increases as the radius of an aneurysm increases (tension = pressure × radius). It is therefore intuitive that larger aneurysms have a greater risk of rupture. Coady et al. [10] have written extensively on the natural history of thoracic aortic aneurysms. Logistic regression analysis of the data revealed a 4.3-fold increased risk of rupture or dissection in an aneurysm 6.0 to 6.9 cm in diameter compared to an aneurysm 4.0 to 4.9 cm in diameter. Growth rates varied from 0.08 cm per year for aneurysms less than 4.0 cm to 0.16 cm per year for aneurysms greater than 8.0 cm in diameter. In the current case, the aneurysm grew the relatively larger size without rupture suggests that some different factors may be effective to lead to rupture besides the radius of the aneurysm. We think that the adhesions which were present in mediastinal and periaortic region secondary to first operation and the long-term use of beta blockers in the current case might prevent the aneurysm to rupture and give rise to the larger diameters.

## 4. Conclusion

We believe that the patients with congenital BAV should be followed regularly in terms of aortic dilatation and aneurysm formation even if their aortic valve is replaced. The surgical management of aortic aneurysm should be done in order to decrease the morbidity and mortality in those patients before the diameter of aneurysm not exceeding relatively larger sizes.

## References

[1] Vallely MP, Semsarian C, Bannon PG (2008). Management of the ascending aorta in patients with bicuspid aortic valve disease. *Heart Lung and Circulation*.

[2] Yasuda H, Nakatani S, Stugaard M (2003). Failure to prevent progressive dilation of ascending aorta by aortic valve replacement in patients with bicuspid aortic valve: comparison with tricuspid aortic valve. *Circulation*.

[3] Agarwal V, Yaliwal C, Ofo E, Kolvekar S (2007). Giant ascending aortic aneurysm—a case report and review. *Heart Lung and Circulation*.

[4] Russo CF, Mazzetti S, Garatti A (2002). Aortic complications after bicuspid aortic valve replacement: long-term results. *Annals of Thoracic Surgery*.

[5] Russo CF, Cannata A, Lanfranconi M, Vitali E, Garatti A, Bonacina E (2008). Is aortic wall degeneration related to bicuspid aortic valve anatomy in patients with valvular disease?. *Journal of Thoracic and Cardiovascular Surgery*.

[6] Matsuyama K, Usui A, Akita T (2005). Natural history of a dilated ascending aorta after aortic valve replacement. *Circulation Journal*.

[7] Borger MA, Preston M, Ivanov J (2004). Should the ascending aorta be replaced more frequently in patients with bicuspid aortic valve disease?. *Journal of Thoracic and Cardiovascular Surgery*.

[8] Bonow RO, Carabello BA, Chatterjee K (2006). ACC/AHA 2006 guidelines for the management of patients with valvular heart disease: a report of the American College of Cardiology/American Heart Association Task Force on Practice Guidelines (Writing Committee to Revise the 1998 Guidelines for the Management of Patients with Valvular Heart Disease)—developed in collaboration with the Society of Cardiovascular Anesthesiologists. *Circulation*.

[9] Nistri S, Sorbo MD, Marin M, Palisi M, Scognamiglio R, Thiene G (1999). Aortic root dilatation in young men with normally functioning bicuspid aortic valves. *Heart*.

[10] Coady MA, Rizzo JA, Goldstein LJ, Elefteriades JA (1999). Natural history, pathogenesis, and etiology of thoracic aortic aneurysms and dissections. *Cardiology Clinics*.

